# Regenerative potential of the intermediate filaments of albino rat parotid glands subjected to fractionated radiotherapy: an immunohistochemical analysis

**DOI:** 10.2340/aos.v84.44182

**Published:** 2025-08-21

**Authors:** Sherif S. Hassan, Ehab T. Azab, Alaa W. AlQutub, Mashael S. Alqahtani, Abrar K. Demyati, Abdullah A. Holdar, Fatma M. Alkassimi, Mahmoud A. Attia, Reda A. Nofal

**Affiliations:** a Department of dental and medical sciences, Faculty of Dentistry, Mutah University, Jordan; b Department of Basic and Clinical Oral Sciences, College of Dental Medicine, Umm Al-Qura University, Makkah, Saudi Arabia; c Department of Oral and Maxillofacial Surgery, College of Dental Medicine, Umm Al-Qura University, Makkah, Saudi Arabia; d Department of Oral and Dental Health, Faculty of Applied Medical Sciences, Al-Baha University, Al-Baha, Saudi Arabia

**Keywords:** Regeneration, parotid gland, cytokeratin, radiotherapy

## Abstract

**Objectives:**

Radiotherapy is a common treatment for head and neck malignancies; however, it frequently affects salivary glands, leading to xerostomia. This study evaluated the effects of radiotherapy on cytokeratin localization in the parotid gland, examining whether changes indicate recovery or progressive damage over a year.

**Methods:**

The study included eight control rats and 16 irradiated rats exposed to 30 Gy of radiation over 6 days. The experiment was conducted from January 2023 to April 2024. Subgroup IIa rats were sacrificed 1 month after radiation exposure, while subgroup IIb rats were sacrificed after 1 year. The parotid gland was prepared for histological and immunohistochemical analysis of intermediate filaments.

**Results:**

In the control parotid gland, immunohistochemical analysis revealed mild cytokeratin in ductal and serous cells. Subgroup IIa exhibited strong cytokeratin expression in the acini and duct cells, which was significantly different from the control group. One year after radiation, the cytokeratin of subgroup IIb was comparable to that of the control, with no significant difference.

**Conclusion:**

In subgroup IIa, cytokeratin staining was notably stronger in ductal and acinar cells, leading to disrupted distribution that impaired saliva production and transport. In subgroup IIb, the redistribution of cytokeratin exhibited distinct recovery patterns in ductal and acinar cells.

## Introduction

Radiotherapy (RT) is a widely used treatment for oral and para-oral malignancies, especially squamous cell carcinoma [1, 2]. It enhances clinical, aesthetic, and functional outcomes as a primary treatment or as an adjunct to surgery to eliminate residual cancerous tissue [3]. Fractionated RT has been the standard treatment plan, often involving the salivary glands within the radiation field [4]. While radiation therapy is essential for tumor treatment, it can also damage the surrounding tissues by exerting toxic effects on both healthy and malignant cells, leading to both short-term effects due to cell death and long-term effects due to vascular and neural damage. Short-term effects on oral and para-oral tissues include dry mouth, oral mucositis, gingivitis, difficulty swallowing, loss of taste, trismus, and opportunistic infections [5]. In contrast, long-term effects may arise over several months, affecting tooth structure, jawbones, vascular integrity, and potentially the periodontium [6–7]. The direct and indirect impact of RT on the periodontium increases the risk of periodontal attachment loss, tooth mobility, and osteoradionecrosis of the alveolar bone, particularly in cases of poor oral hygiene.

The parotid gland (PG) is the largest major salivary gland in most mammals, produces approximately 23% of saliva, and is essential for oral health, contributing to digestion, taste perception, speech, antimicrobial defense, and maintaining oral and dental integrity [8–11]. Shielding the tissues surrounding the cancer from radiation exposure is challenging, often leading to considerable damage, especially to the salivary glands. This damage hinders saliva production and flow, ultimately leading to xerostomia [13]. Dry mouth is the most damaging short- and long-term side effect of radiation, as decreased saliva production contributes significantly to oral mucositis, tooth decay, and periodontal diseases [13–15].

The cytoplasm of a cell contains a three-dimensional network of filaments known as the cytoskeleton, which is composed of three main elements: microtubules (25 µm in diameter), intermediate filaments (IF, 6–10 µm), and microfilaments (4–6 µm). In animal cells, six major types of IFs have been identified: cytokeratin, vimentin, desmin, glial fibrillary acidic protein (GFAP), neurofilaments, and nuclear lamins [16–17]. Cytokeratin IF is a family of proteins found in epithelial cells, where they help maintain cell structure, connect adjacent cells, support the movement of cytoplasmic organelles, assist in intracellular transport, and contribute significantly to the tensile strength and structural integrity of epithelial tissues [1]. Cytokeratin 17 (CK17) is a type of IF found in ectodermal acinar and ductal cells of the salivary gland parenchymal elements, playing a key role in supporting the intracellular transport of raw materials and secretory products [1, 17]. Mapping IF in cells is important due to their critical roles in indicating the cell differentiation and identity, maintaining cellular integrity and function, aiding in disease diagnostics and biomarker discovery of cancer, and early detection of diseases. Additionally, understanding the distri-bution of IF relative to the nucleus and organelles is essential for studying cellular organization and intracellular transport [18].

Immunohistochemistry (IHC) is a staining method that detects CK17 using an antigen-antibody reaction to localize the target molecule expression within its specific environments [19]. IgG is a commonly used antibody in IHC, produced by immunizing an animal with a purified specific antigen that serves as an immunogen. This process triggers humoral responses, forming a monoclonal antibody (anti-epitope) that is subsequently isolated from the animal and used to identify antigen expression in the targeted cells. CK17 is a valuable biomarker because of its resistance to degradation, strong antigenicity, stability, and reliable expression patterns in formalin-fixed, paraffin-embedded tissues.

Salivary gland healing after radiation injury is dose-dependent and involves a coordinated process with different cellular and molecular mechanisms working together to restore glandular function and tissue integrity [16]. Many researchers have investigated strategies to prevent or reverse radiation-induced salivary gland damage, with the goal of rapidly restoring saliva production after RT. Li et al. reported that the functional regeneration of radiation-damaged salivary glands can be achieved through the sustained delivery of the synthetic neurogenic muscarinic receptor agonist [20]. It is still unclear whether acinar cells can undergo cell division in response to muscarinic stimulation or regenerate into functional acinar cells. Chibly et al. observed that regenerative treatments for irradiated PG are currently unavailable to restore their acinar cell population or repair organ function [21]. Furthermore, the authors noted that existing treatment options, including saliva stimulants, saliva diuretics, and saliva substitutes, provide only palliative relief and do not restore the roles of the salivary glands.

Recent studies have identified a small population of stem cells within salivary gland tissues linked to the duct system, which has attracted considerable interest in regenerative medicine due to their potential to repair damaged glandular tissue [22]. According to Song et al., salivary gland tissue contains stem cells, including embryonic stem cells and induced pluripotent stem cells, which are promising for cell therapies due to their unlimited proliferation and differentiation capacity [6]. Aure et al. observed that salivary gland damage can be repaired by replicating functional acinar and ductal cells or differentiating stem cells [23].

Our previous studies have demonstrated variations in IHC between radiated and non-radiated salivary glands at doses of 25–30 Grays (Gy), either one shot or fractionated, with stronger expression observed in the radiated glands [12, 14, 18]. Hassan and Qahtani [13] reported that male albino rats exposed to a total of 30 Gy of RT (5 Gy per day) targeting the major salivary glands exhibited increased CK17 expression in duct cells and some serous acini, while mucous acini remained negative. Compared to the mild expression observed in the control group, the irradiated glands showed an altered distribution pattern, indicating pathological changes within a month post-irradiation [12]. The same researchers investigated the effects of radiation on the submandibular gland at 1 month and 1 year post-exposure. They found that in irradiated samples, serous acinar cells exhibited moderate to strong CK17 expression in ductal cells compared to the control group. However, by the year, acinar cells in the third group showed CK17 expression levels comparable to those of the control group. This indicates a potential natural recovery of the gland’s cellular composition without medical intervention [24]. The present study aims to assess the effects of RT on the mapping of IF, comparing changes 1 month and 1 year after the final dose to determine whether the results indicate gland recovery or continued destruction.

## Materials and methods

### Animal grouping

The study included 24 albino rats of approximately 170 grams. The rats were kept in the Laboratory House for Animals at the Faculty of Veterinary Medicine, Cairo University, Giza. They were provided with hard and soft foods with free access to water. The rats were divided into eight control group I and 16 irradiated group II. The irradiated rats were exposed to a total radiation of 30 Gy, administered over 6 consecutive days at a rate of 5 Gy per day. Group II was subdivided into two subgroups: Subgroup IIa rats were sacrificed 1 month after stopping the radiation, and subgroup IIb rats were allowed to survive for 1 year before being sacrificed.

### Radiotherapy exposure

During each day of RT, all rats, including those in group I, were anesthetized using thiopental sodium 30 mg/kg (Pharmaceutical Co., Egypt). The rats of the second group were exposed to a total radiation dose of 30 Gy, which is equivalent to the lowest fractional RT dose to which a human is exposed [24]. A radiation dose of 5 Gy was delivered at a rate of 1 Gy per minute to a 25 × 25 mm field targeting the salivary gland complex, with a 6 mm thick lead layer used to shield the surrounding vital organs from exposure. Treatment was administered between 09:00 and 13:00 using a therapeutic X-ray [Philips SL;75.5]. The X-ray machine operated at 235 kV and 15 mA, with a focus-to-surface distance of 43 cm.

### Tissue preparation

At the designated time for the experiment, the rats were euthanized, and the PG was meticulously dissected out and fixed in 10% neutral formalin for 3 days. Following fixation, the tissues were embedded in paraffin and prepared for staining with Hematoxylin & Eosin for standard histological examination.

### Immunohistochemical processing

Tissue sections (5–6 μm) were mounted on silicone-coated slides, deparaffinized with xylene, and rehydrated through graded ethanol. Endogenous peroxidase activity was blocked using 0.3% H₂O₂ in methanol. Antigen retrieval was performed by microwave heating, followed by a 10-min incubation in blocking solution. CK17 expression was detected using the monoclonal anti-CK17 E3 antibody via the LSAB method, with hematoxylin counterstaining. Positive CK17 staining appeared brown in both acinar and ductal cells and was scored from 1 (weak) to 4 (strong).

### Statistical data analysis

Data were written and calculated to be analyzed through the Statistical Package for Social Science (SPSS), version 23 (IBM Inc., United States). A statistical analysis was conducted to compare CK17 expression levels among non-irradiated and irradiated subgroups. The analysis started with calculating the statistical summary for each group, covering the mean, median, standard deviation, and range, and creating a histogram to visualize data distribution. A one-way analysis of variance test was done comparing the results between groups, and both Least Significant Difference (LSD) and Dunnett’s tests were used to assess significant differences in mean values between groups. *P*-value was considered significant at the level of ≤0.05.

## Results

During the radiation period, five rats from different groups died within 24 h of the first day of radiation exposure, likely due to complications from anesthesia. Dead rats were excluded from the experiment and replaced by five others who underwent a radiation protocol the following week to complete the number of rats to be irradiated.

### Histopathological evaluation

**Figure 1:**
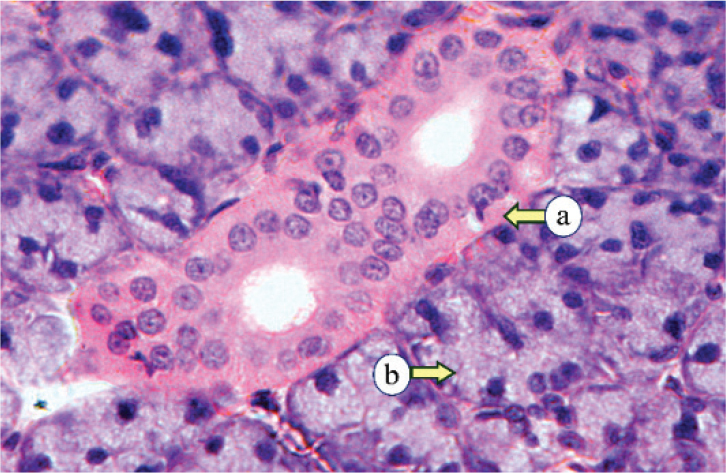
Control parotid glands displaying the intralobular ducts (a) and rounded serous acini (b) (H&E, × 400).

**Figure 2:**
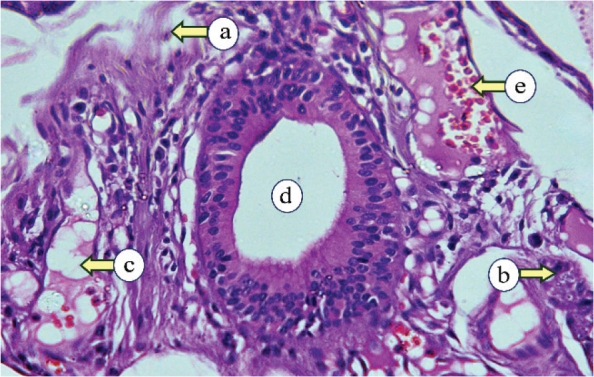
Subgroup IIa, examined 1-month post-irradiation, showing fibrous tissue (a), degenerated acini (b), fatty tissue (c), dilated duct (d), and extravasated blood (e) (H&E, × 400).

**Figure 3:**
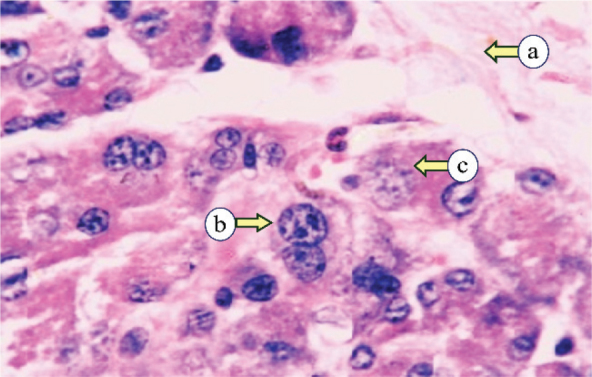
Subgroup IIb, examined 1-year post-irradiation, revealing fibrous tissue (a), mitotic figures (b), and enlarged cells with scanty cytoplasm (c) (H&E, × 400).

Histopathological examination of the control PG revealed numerous lobular parenchymal tissues with densely packed serous acini and intercalated and striated ducts, all supported by connective tissue stroma that divided the gland into distinct lobes and lobules (Figure 1). The PG of subgroup IIa showed atrophic changes, marked by a reduction in the acinar and ductal elements with a condensed fibrous tissue stroma. The acini were smaller, with reduced serous cells and indistinct lumens. All ducts were observed to be dilated, encircled by remnants of acinar cells, and interspersed with scattered duct-like structures (Figure 2). The PG of subgroup IIb exhibited two distinct histological changes, each with a different appearance. The first type revealed atrophy of the parotid tissue, where fibrous tissue had replaced the normal structure, and the persisting acini were smaller and further apart. The 2nd type demonstrated proliferative activity, with enlarged acini, numerous mitotic figures, and fibrous tissue with small, persistent acini (Figure 3).

### Immunohistochemical analysis

**Figure 4:**
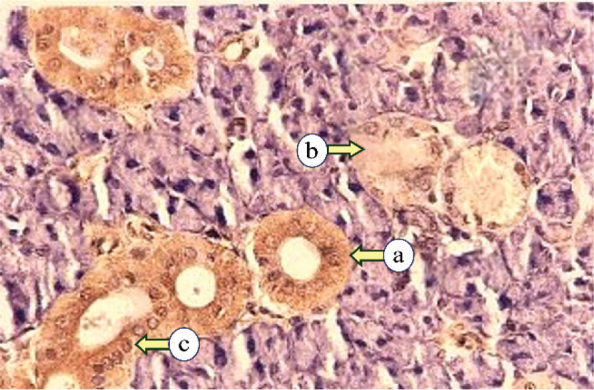
Photomicrographs of the control parotid glands displaying mild expression of Cytokeratin17 in the striated duct (a), intercalated duct (b), and serous acini (c) (Immune-peroxidase technique, × 200).

Sections from the control group demonstrated mild expression in various duct and serous cells, following two distinct distribution patterns. The first pattern exhibited diffuse, uniform expression throughout the cytoplasm. The second pattern showed expression concentrated at the basal cytoplasmic region, with weak expression near the lumen. The excretory duct showed moderate staining in the basal cells and diminished in the superficial layers (Figure 4).

**Figure 5:**
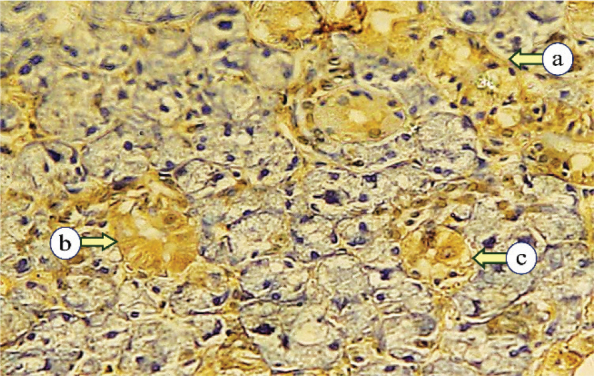
Photomicrograph of subgroup IIa, showing strong diffuse Cytokeratin 17 expression in the intercalated duct (a), mild to moderate expression in serous acini (b), and strong at the luminal part with mild staining at the basal part of the striated duct (c) (Immune-peroxidase technique, ×200).

In irradiated subgroup IIa, CK17 expressions ranged from mild to severe in the intralobular ducts. The staining pattern was diffused or focused in the luminal cytoplasmic region. Some excretory ducts exhibited severe expression, either concentrated at the apical portion of the cells with mild expression basally or diffusely distributed throughout the cells. Various serous acini exhibited moderate diffuse expressions, while some degenerated acini and ducts showed mild cytokeratin expression (Figure 5).

**Figure 6:**
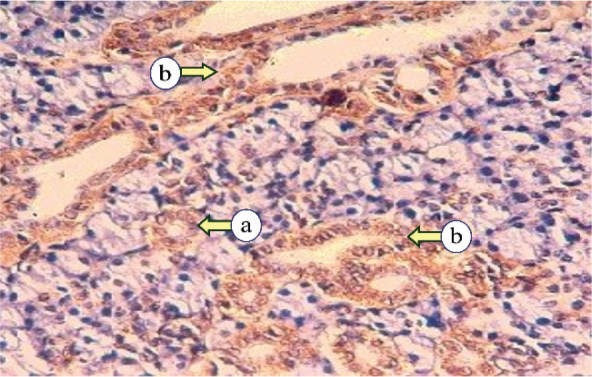
Photomicrograph of subgroup IIb, revealing mild Cytokeratin 17 expression of serous acini (a), and moderate cytokeratin expression of striated ducts (b) (Immune-peroxidase technique, × 200).

In irradiated subgroup IIb, CK17 expression ranged from mild to strong in duct and serous cells, with most cases exhibiting diffuse cytoplasmic distribution. The staining was localized to the luminal cytoplasmic region in a few specimens, with mild staining in the basal portion. Additionally, moderate expression was noted in some excretory ducts and degenerated acini (Figure 6).

### Statistical results

**Figure 7:**
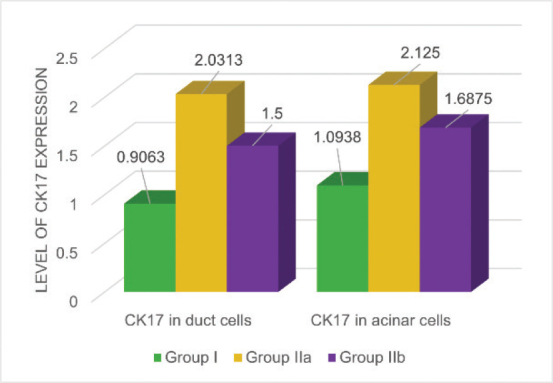
Cytokeratin expression in the various groups.

The statistical summary of all variables, including the mean, median, standard deviation, and range, along with the corresponding histogram, is presented in Table 1 and Figure 7. The staining of CK17 in the duct cells among the control and radiated groups was significantly higher than in group I (*P* = 0.006, Table 2). Although CK17 expression in the duct cells of subgroup IIb was higher than in group I, with no statistical significance based on the LSD test (*P* = 0.069, Table 3). Additionally, subgroup IIa exhibited lower CK17 expression than subgroup IIb. However, this difference was nonsignificant according to the Dunnett test. ANOVA test revealed significant differences in CK17 staining levels among acinar cells across all groups (*P* = 0.029, Table 2). However, the Dunnett test showed that subgroup IIb had significantly lower CK17 expression than subgroup IIa (*p* > 0.05), suggesting that prolonged duration may reduce cytokeratin intensity.

**Table 1:** Descriptive analysis of CK17 expression in the parotid gland parenchymal cells across all groups.

(CK17 intensity)	Group	*N*	Mean	Std. deviation	Std. error mean	95% confidence interval for mean		Minimum	Maximum
						Lower bound	Upper bound		
Staining of CK17 in the parotid gland duct cells	Group I	8	0.9063	0.51647	0.18260	0.4745	1.3380	0.50	2.00
	Group IIa	8	2.0313	0.86021	0.30413	1.3121	2.7504	1.00	3.50
	Group IIb	8	1.5000	0.37796	0.13363	1.1840	1.8160	1.00	2.00
	Total	24	1.4792	0.75512	0.15414	1.1603	1.7980	0.50	3.50
Staining of CK17 in the parotid gland acinar cells	Group I	8	1.0938	0.80109	0.28323	0.4240	1.7635	0.25	2.50
	Group IIa	8	2.1250	0.61237	0.21651	1.6130	2.6370	1.25	3.00
	Group IIb	8	1.6875	0.71651	0.25333	1.0885	2.2865	0.75	3.00
	Total	24	1.6354	0.80750	0.16483	1.2944	1.9764	0.25	3.00

**Table 2:** Test of Analysis of variance (ANOVA) on CK17 expression in the parotid gland parenchymal cells across all groups.

(CK17 intensity)	(Inter-compare)	Sum of squares	df	Mean square	*F*	Sig.
Staining of CK17 in the parotid gland duct cells	Between Groups	5.068	2	2.534	6.613	0.006
	Within Groups	8.047	21	0.383		
	Total	13.115	23			
Staining of CK17 in the parotid gland acinar cells	Between Groups	4.286	2	2.143	4.202	0.029
	Within Groups	10.711	21	0.510		
	Total	14.997	23			

**Table 3:** Multiple comparisons LSD and Dunnett test (2-sided) on CK17 expression in the parotid gland across groups.

(CK17 intensity)		(I) Groups	(J) Groups	Mean difference (I-J)	Std. error	Sig.	95% confidence interval	
							Lower bound	Upper bound
Cytokeratin 17 expression of duct cells	LSD	Group 1	Group IIa	−1.12500^*^	0.30951	0.002	−1.7687	−0.4813
			Group IIb	−0.59375	0.30951	0.069	−1.2374	0.0499
		Group IIa	Group 1	1.12500^*^	0.30951	0.002	0.4813	1.7687
			Group IIb	0.53125	0.30951	0.101	−0.1124	1.1749
		Group IIb	group 1	0.59375	0.30951	0.069	−0.0499	1.2374
			Group IIa	−0.53125	0.30951	0.101	−1.1749	0.1124
	Dunnett t (2-sided)a	Group 1	Group IIb	−0.59375	0.30951	0.121	−1.3274	0.1399
		Group IIa	Group IIb	0.53125	0.30951	0.175	−0.2024	1.2649
Cytokeratin 17 expression of acinar cells	LSD	Group 1	Group IIa	−1.03125^*^	0.35709	0.009	−1.7739	−0.2886
			Group IIb	−0.59375	0.35709	0.111	−1.3364	0.1489
		Group IIa	Group 1	1.03125^*^	0.35709	0.009	.2886	1.7739
			Group IIb	0.43750	0.35709	0.234	−0.3051	1.1801
		Group IIb	Group 1	0.59375	0.35709	0.111	−0.1489	1.3364
			Group IIa	−0.43750	0.35709	0.234	−1.1801	0.3051
	Dunnett t (2-sided)a	Group 1	Group IIb	−0.59375	0.35709	0.192	−1.4401	0.2526
		Group IIa	Group IIb	0.43750	0.35709	0.381	−0.4089	1.2839

* The mean difference is statistically significant at the 0.05 level.

** The Dunnett test treats one group and compares the other groups against it.

## Discussion

Fractionated RT is employed to minimize the complications associated with radiation treatment. When applied to the head and neck region, it helps reduce adverse effects; however, despite the use of fractionated doses, significant oral complications may still occur. Fractionated RT for head and neck cancers often leads to oral complications like mucositis, taste loss, and xerostomia, though the mechanisms underlying radiation-induced salivary gland damage remain unclear [2–5]. This study aimed to evaluate the PG injuries resulting from RT and their ability to recover and regain normal function over time, provided that cancer treatment is fully completed.

Our results indicate that the PG exhibited persistent atrophy with fibrous tissue replacement 1 year after radiation cessation, emphasizing the long-term effects of radiation observed in conventional histopathological analysis. These findings are consistent with numerous studies reporting that persistent pathological damage to the salivary glands occurs in about 90% of patients exposed to radiation, especially at doses above 30 Gy [12, 25]. The observation of numerous smaller acini in our results after RT suggests that the gland maintains its secretory function, albeit at a diminished level. According to various studies, this adaptation enables acinar cells to shift into a resting state with decreased secretory activity until the pathological threat subsides [26, 27].

In this study, numerous irradiated gland specimens from the 1-year subgroup IIb exhibited large cells with mildly vacuolated cytoplasm and numerous mitotic figures. These findings may suggest either the beginning of glandular healing or abnormal mass formation, aligning with observations reported by various authors [28, 29]. Emmerson et al. also highlighted that certain progenitor cell populations require time to contribute to the regenerative capacity of salivary glands in response to radiation-induced damage [16].

Our results showed that the control PGs exhibited stronger CK17 in the duct cells than in the serous cells. This indicates that the acinar cells are highly differentiated, with a lower expression of IFs to facilitate the passage of salivary secretion for exocytosis. The varying CK17 expression patterns observed in the control glands likely indicate glandular activity, where a diffuse pattern suggests a resting state, while decreased expression in the apical region is linked to exocytosis [30]. The second expression pattern was concentrated in the basal cell region, possibly related to enhanced tensile strength in acinar cells adjacent to the contractile myoepithelial cells. This might improve the pressure capacity, increasing saliva flow through the lumen into the duct [1].

Group IIa showed markedly stronger CK17 staining in the ductal and serous cells compared to the control group. This enhanced expression may be attributed to the disruption and aggregation of IFs within the cytoplasm, potentially impairing saliva production and the exocytosis transport pathway. Two patterns of cytokeratin expression were observed: one displayed a diffuse distribution throughout the entire cell, potentially indicating extensive cellular damage, while the other exhibited localized expression at the luminal region, which may impede exocytosis and disrupt saliva modification within the duct system [12].

Numerous studies have explored the regeneration of irradiated salivary glands, with some focusing on stem cell-based therapies, others examining the nervous system’s role in gland activation, and some investigating palliative medical treatments aimed at symptom relief [16, 31]. However, the current research adopted a different approach by examining the potential for natural healing without medical interventions, emphasizing the gradual redistribution of CK17 within the cell toward its normal state over time. The statistical results for subgroup IIb indicate a slow recovery of ductal and acinar cells, supporting the potential for recovery responses observed 1 year after radiation cessation and underscoring the need for a prolonged recovery period. While the sustained significance in ductal cells across irradiated subgroups compared with the control group confirms the lasting impact, the reduced expression of CK17 in the statistical data of group IIb indicates that ductal cell recovery may have initiated but remains insignificant. The statistical analysis of acinar cells suggested no significant difference between subgroup B and the control group, indicating that acinar cells underwent more advanced recovery and emphasizing the differing injury responses and repair timelines relative to ductal cells. This accelerated recovery of acinar cells highlights their essential role in resuming saliva secretion.

Underwent more advanced recovery and emphasizes the differing injury responses and repair timelines relative to ductal cells.

## Conclusions

The hypothesis of the experiment is based on the observation that IF expression, when comparable to that of the control group, suggests cellular recovery. Therefore, both biological and clinical approaches are necessary to evaluate saliva secretion rates and analyze the chemical composition of the saliva to confirm this recovery. The expression of CK17 showed distinct patterns in control versus irradiated PGs, suggesting a correlation between CK17 distribution and glandular function. The recovery of ductal and acinar cells followed separate trajectories, with acinar cells demonstrating more advanced healing. This highlights the differing responses to injury and varied repair timelines between ductal and acinar cell types.
